# Healthcare practitioner views and experiences of patients self-monitoring blood pressure: a vignette study

**DOI:** 10.3399/bjgpopen20X101101

**Published:** 2020-11-11

**Authors:** Jacob A Andrews, Kate Weiner, Catherine M Will, Flis Henwood, Jon M Dickson

**Affiliations:** 1 Department of Sociological Studies, University of Sheffield, Sheffield, UK; 2 Division of Psychiatry and Applied Psychology, School of Medicine, University of Nottingham, Nottingham, UK; 3 School of Law, Politics and Sociology, University of Sussex, Brighton, UK; 4 School of Applied Social Science, University of Brighton, Brighton, UK; 5 Academic Unit of Primary Medical Care, University of Sheffield, Sheffield, UK

**Keywords:** focus groups, general practitioners, hypertension, practice nurses, primary health care, qualitative methodology, self-monitoring, vignettes

## Abstract

**Background:**

Home self-monitoring of blood pressure is widely used in primary care to assist in the diagnosis of hypertension, as well as to improve clinical outcomes and support adherence to medication. The National Institute for Health and Care Excellence (NICE) care pathways for hypertension recommend specific guidelines, although they lack detail on supporting patients to self-monitor.

**Aim:**

To elicit primary care practitioners’ experiences of managing patients’ home blood pressure self-monitoring, across surgeries located in different socioeconomic areas.

**Design & setting:**

A qualitative focus group study was conducted with a total of 21 primary care professionals.

**Method:**

Participants were GPs and practice nurses (PNs), purposively recruited from surgeries in areas of low and high deprivation, according to the English indices of multiple deprivation. Six vignettes were developed featuring data from interviews with people who self-monitor and these were used in five focus groups. Results were thematically analysed.

**Results:**

Themes derived in the thematic analysis largely reflected topics covered by the vignettes. These included: advice on purchase of a device; supporting home monitoring; mitigating patient anxiety experienced as a result of home monitoring; valuing patients’ data; and effect of socioeconomic factors.

**Conclusion:**

The work provides an account of methods used by primary care practitioners in the management of home blood pressure self-monitoring, where guidance may be lacking and primary care practitioners act on their own judgement. Findings complement recent policy documentation, which recognises the need to adopt new ways of working to empower patients (for example, additional support from healthcare assistants), but lacks detail on how this should be done.

## How this fits in

Policy documents, including the NHS Long Term plan, advocate for the increased use of technology in healthcare pathways across the NHS. Blood pressure self-monitoring has been conducted by patients in their own homes for many years, and thus provides a case from which lessons may be learnt on a variety of aspects of management of patient-owned technologies mobilised in primary care. The study provides an account of methods used by primary care practitioners in the management of home blood pressure self-monitoring, where guidance may be lacking and primary care practitioners act on their own judgement.

## Introduction

The self-monitoring of blood pressure at home was introduced in the 1930s^1^, and is associated with improved clinical outcomes in hypertension, when accompanied by appropriate interventions.^2–4^ Since 2011,^5^ NICE has advocated home blood pressure monitoring as one method of assisting the diagnosis of hypertension, and continues to issue guidance on the use of this method in the updated (2019) guidelines.^6^ Now, home self-monitoring of blood pressure is widely used in primary care to assist in the diagnosis of hypertension, although adherence to NICE guidelines is known to vary.^7^ Furthermore, the guidelines do not provide detail on supporting patients to self-monitor, for example, no detail is given regarding advice on purchase or use of a monitor.^5,6^

Qualitative work exploring blood pressure self-monitoring has, to date, mostly been conducted within the confines of randomised controlled trials, where self-monitoring has been directed as part of a research protocol.^8^ There is a need to explore the management of self-monitoring in the everyday work of primary care, where practitioners act on their own discretion and where practice is likely to vary. Smolen and colleagues have developed a conceptual model of hypertension management in general practice, identifying three main strategies used by providers to support patients: education (about the condition and its consequences); relationship building (relating to continuity of care); and use of self-management tools (including home monitors).^9^ Their study and other prior work focuses largely on managing care once a patient has obtained a monitor, excluding guidance provided prior to this point.^8,9^ Some work has additionally explored the potential of new technologies (for example, smartphone apps) for measuring blood pressure in primary care,^10^ although not considering this within the context of current everyday practice. In terms of guidance, patients are sometimes unconfident in the use of home monitors, and may seek information online.^4^ Healthcare assistants may be involved in providing support and guidance to patients who self-monitor, although research into their role is limited.^11^

Funded by a 3-year Leverhulme Trust research grant, the *Tracking Ourselves?* Project^12^ has explored everyday experiences of self-monitoring, through interviews with people who had acquired a device either on recommendation of a clinician or out of their own interest. Finding consistent references to primary care in many of these interviews, the study sought to further explore the relationship between primary care professionals and patients who self-monitor blood pressure by conducting focus groups with GPs and PNs. Discussions focused on routine daily practice and professional experience.

## Method

### Aim

The study aimed to elicit primary care practitioners’ views and experiences of managing patients’ home blood pressure self-monitoring.

### Recruitment

Primary care health professionals were recruited with assistance from the National Institute for Health Research’s (NIHR) Clinical Research Network (CRN) in Yorkshire and Humber, UK. GP practices and participants were contacted via two CRN clusters (groups of research-active GP practices). Six practices and one research group were approached, although two practices were unable to participate. The authors purposively recruited from surgeries in both higher and lower socioeconomic areas, to explore differences in views and practice across primary care practitioners working in these areas. Healthcare professionals were eligible to take part if they had experience working as a GP or PN in general practice. Written informed consent was obtained from all participants.

In total, five focus groups were conducted, with a total of 21 health professionals, from two practices in higher socioeconomic areas, two practices in lower socioeconomic areas, and one group formed of practitioners from a cluster based in lower socioeconomic areas. The English Indices of Deprivation 2015 was used to determine socioeconomic status.^13^

### Methodology

Vignettes were developed to structure the group discussions. Vignette methodology involves the use of short sections of text describing a person or situation to prompt a discussion between the researcher(s) and the participant(s).^14^ Vignettes have been used in surveys,^15–17^ interviews^18^ and focus groups,^19^ and their content is usually hypothetical.

The research discussed in this article is part of a larger project exploring how and why people self-monitor, involving interviews with 84 people who monitor their own blood pressure and/or body mass index (BMI). The authors drew on analysis from these interviews to design vignettes that would stimulate discussion among healthcare professionals, selecting excerpts that provided good illustrations of common experience. The vignettes focused on multiple points of care: diagnosis; obtaining a monitor; use and storage of blood pressure data in clinic; self-management of medication; and patient anxiety. Vignettes were refined through piloting with local clinical and academic staff. In the focus groups, participants considered the scenarios presented in the vignettes and reflected on their own experiences.

### Procedure

Of the five focus groups, four took place in rooms at the GP surgeries where participants were employed, and one was held in a university building at the research meeting of one of the clusters. Each focus group involved discussion of the same six vignettes.

After taking informed consent, the researchers read out each vignette before asking an opening question to prompt discussion. Each 1-hour focus group then followed a semi-structured approach, using a question guide prepared in advance (see supplementary files). Two researchers were present at each focus group, except for one focus group (FG5) where only one researcher was able to attend. Both researchers took notes when present. Sessions were audio-recorded.

### Analysis

Audio-recordings were transcribed verbatim and analysed using thematic analysis.^19^ JA led the analysis, while KW and CW analysed a subsample of the data (one focus group) and discussed the development of initial themes with JA. Final themes were derived through an iterative analysis of all focus group transcripts, in line with Braun and Clarke’s methodology.^19^ In the quotes below, participants are labelled by their focus group number (‘FG#’), then by their role in general practice (GP or PN), then by their participant number within each focus group, for example, ‘FG3GP2’. The letter in brackets dictates the participant’s sex.

## Results

### Participants

Five focus groups were conducted, with a total of 21 general practice clinicians. These consisted of 14 GPs and seven PNs. Focus groups ranged in size from two to eight participants. Of the 21 participants, 14 were female (66%). Years of experience in general practice ranged from 1–24 years, with a median of 8 years. Participant demographics are presented in Supplementary Table S1 and Supplementary Table S2.

### Thematic analysis

Themes mostly reflected the focus of the vignettes that were developed; however, some discussions on particular vignettes cut across themes, and vice versa. For example, quotes in the theme on valuing patients’ self-monitoring data occurred in reference to both Frank and Emily’s vignettes. The final themes were: ‘advice on purchase of a device’; ‘supporting home monitoring’; ‘mitigating patient anxiety experienced as a result of home monitoring’; ‘valuing patients’ self-monitoring data’; and ‘effect of socioeconomic factors’. Each of these is presented below with excerpts from the focus groups to demonstrate the main findings.

#### Advice on purchase of a device

One vignette described how an interviewee had been given advice by her GP on which blood pressure monitor she should buy on Amazon. Many participants were shocked that this had occurred (for example, one participant stated: *'I think it's morally questionable*' [FG1GP6{F}]), and discussed how they would advise patients on what to buy and where to source it from.

Some were happy to recommend purchasing a device from a specific pharmacy, including the one that was attached to their surgery:

*'I think we can rely on* [Harry] *and the team next door* [in the pharmacy] *to make sure they got a quality approved one, for a good price, with instructions of how to use it*.' (FG2GP2[M])

For others, it was considered unethical to make any recommendations at all:

*'We’re not meant to recommend a pharmacist to have their medications sent to, are we? So you sort of do feel a bit that you can’t over-recommend things*.' (FG3GP2[F])

Several participants suggested they would recommend their patients to consult the British Hypertension Society’s list of recommended devices:

*'* *I think you can get British Hypertension Society, you know, approved blood pressure monitors, so* [I would recommend] *one of them* *.'* (FG1PN2[F])

#### Supporting home monitoring

The theme on supporting home monitoring included two subthemes. First, this concerned methods that practitioners had used to educate patients on the use of a home blood pressure monitor. These included sending links to NHS Choices (now referred to as the NHS website) via SMS, and referring patients to YouTube, as well as setting up appointments with healthcare assistants for face-to-face instruction on how to use a monitor:

*'There’s lots of really good stuff on YouTube from very reputable sources* [...] *they can go back and look at it again, rather than me explaining it and you think, “oh, I've done a great job there”, and off they go and they're like, “what do I do with this?”'* (FG1GP5[M])

*'I use SMS. So, we’re on SystmOne and it’s set up so you can send people SMS and I quite often send them a link to NHS Choices*.' (FG5GP2[F])

Second, participants discussed how patients had brought their blood pressure monitor to the surgery in order to test its accuracy against the surgery monitor. They related their experience to Frank’s vignette, which described how Frank had been advised by his GP to measure his blood pressure at home, and it was no longer being measured in the clinic:

*'I think one thing I would want to know is that the machine he was using had been validated, so the first time I would get him to come and see the healthcare assistant, say, “bring your machine in, we’ll check it on yours, check it on ours and make sure it's the same and then off you go and maybe we would do that every 12 months*.”' (FG1GP3[M])

However, there were mixed views on the value of patients bringing their monitor for calibration, with some GPs finding it unnecessary given the accuracy of blood pressure monitors overall. Some GPs were unaware that this was being conducted by colleagues in other roles:

*'I think I sigh when somebody brings a blood pressure monitor in to me, inwardly sigh and think, “oh really?”* [...] *You have to take a ball park I think really with it,* [...] *if I did ten blood pressures with my monitor consecutively, they’re all going to be different*.' (FG4GP1[M])

*'I don’t think we’ve ever done that, have we?* [...]*'* (FG5GP2[F])*‘Some do* *.'* (FG5PN1[F])*‘Do they?* *'* (FG5GP1[M])

Several participants saw it as the healthcare assistant’s role to support home monitoring and take blood pressures in the surgery where required, allowing GPs to concentrate on the analysis of data and decision making around care:

*'A lot of these problems that have been identified are the sort of things that with a well-trained healthcare assistant they could sort that out*.' (FG1PN1[F])

#### Mitigating patient anxiety experienced as a result of home monitoring

One vignette focused on the case of a patient (Gary) who had been advised to monitor his blood pressure less often because he had health anxiety. The participants were unanimous in backing the decision of his GP, and recognised patients like this in their own practice:

*'Gary may need some other type of help to manage his health anxiety, but monitoring his blood pressure all the time, in my opinion, is not going to do him any favours.'* (FG4GP1[M])

One way of mitigating the anxiety caused by self-monitoring was to share guidelines on how often to monitor with the patient, as a way to reassure them that it was safe not to monitor on a frequent basis:

*'There’s fairly clear guidelines on how frequently people should have blood pressure checks, which I might, kind of, share with him as a way of reassuring him.'* (FG4GP2[M])

Other practitioners used the offer of ambulatory monitoring as a way to take pressure off patients and reduce their level of anxiety:

*'It’s on for 24 hours and then you can forget about it, he’s not got the added anxiety of, “oh, I’ve got to take my blood pressure” and, “oh, what’s it going to be and am I doing it right?”'* (FG2PN1[F])

Participants also advocated emphasising the importance of a longer-term approach to blood pressure with more anxious patients to mitigate concern:

*'It’s important and we need to do it properly, but we need to do it as a routine matter, this is not something that you need to get worried or stressed about and it’s something we need to sort out over months and years*.' (FG2GP1[M])

#### Valuing patients’ self-monitoring data

Another vignette presented the case of Emily, who had remarked that her GP had not recorded the blood pressure readings she took with her to the surgery. Participants discussed the importance of making obvious to the patient why they are collecting data, recognising that patients may be unclear about how their data is used:

*'She’s* [Emily] [...] *not sure whether or not the doctor is taking it seriously or really cares. I hope my patients don’t think that, I think I tell them the conclusion I’ve drawn*.' (FG2GP1[M])

Some reflected that this may be a blind spot in the way they work:

*'I think this touches on a really important thing though and I think I'm really bad at this actually* [...] *I don't value the work and the time they have put into producing this information* [...] *it's not going to encourage them to carry on doing it*.' (FG1PN2[F])

Some followed particular ways of working in relation to blood pressure records that patients had brought, but were unsure whether the data were processed in line with those policies:

*'So, people just tend to bring in the sheets we give them, and I do give those to the receptionists to scan in. I think we scan them in. I’ve never checked*.' (FG5GP1[M])

#### Effect of socioeconomic factors

The authors purposively sampled from practices in areas of higher and lower deprivation according to the English Indices of Deprivation. In practices in more deprived areas, clinicians reported that they might recommend their patients to ask family and friends if they could borrow a blood pressure monitor, or lend them one from the surgery, rather than advising patients to buy their own:

*'Income is an issue. This is a fairly deprived practice. So, sometimes you end up having conversations about, has your mum got one, do you know someone who’s got one, as well*.' (FG5GP2[F])

One of the vignettes described the case of Bob, a tiler who reported ‘confessing’ to his GP that he had taken less medication than instructed. Reacting to this vignette, participants in higher socioeconomic areas commented that they did not find their patients to be deferential or to express guilt about querying instructions from healthcare professionals, and expressed their assumption that they were more used to patients directing their own monitoring or medication:

*'* *It’s a very, very middle class area, so they ask a lot more questions.* *'* (FG3GP1[F])

*'* *It’s very educated.* *'* (FG3GP2[F])

*'* *A lot less deferential* *.'* (FG3GP1[F])

*'So our patient demographic is* [...] *skewed towards the higher socioeconomic end of the spectrum, I guess, so we’ve got loads of professionals and, you know, medics and lawyers and teachers* [...] *there’s lots of educated people* [...] *I encounter quite a few people who want to be quite autonomous in how they do these things and I’m quite happy for them to do that*.' (FG4GP2[M])

## Discussion

### Summary

This study has explored primary care practitioners’ experiences and practices in supporting blood pressure self-monitoring in the management of hypertension. The findings demonstrate how clinicians guide patients to make choices about purchasing healthcare technologies, and make clear the need for balance between maintaining professional impartiality and providing patients with guidance to ensure they purchase appropriate devices. Participants indicated pharmacies as reputable vendors of approved blood pressure monitors, and lists of approved devices as resources to which they refer their patients. On educating patients about their devices, several participants stated that they use email or SMS to refer patients to online materials, including videos on YouTube, when these originate from reputable sources. They also refer patients to healthcare assistants to learn how to use blood pressure monitors.

The findings highlight a recognition among primary care professionals of the anxiety patients may experience as a result of home monitoring, and a variety of approaches to mitigate it. These included use of ambulatory monitors to reduce outputs visible to patients that may cause them concern, or providing patients with guidance on monitoring frequency. Participants suggested they were aware of the need to be open with patients about how their self-generated data would be used. This openness was recognised as a way to encourage continued monitoring, and to communicate to patients that primary care professionals value the data that patients provide.

Cost was highlighted as a barrier to some patients purchasing monitors and suggestions were given for overcoming this barrier, for example, patients might borrow a monitor from friends or family, or use one owned by the surgery. The results demonstrate too the possibility of variations in patients’ attitudes to discussing monitoring and blood pressure medication with their primary care practitioner.

### Strengths and limitations

The use of vignettes developed from interviews with people who self-monitor provided authenticity to the study materials. In contrast to traditional vignette methodology, this approach may reduce research participants’ sense that the research is contrived.^18^ The use of vignettes more generally provided a useful framework within which to promote discussion, as indicated by Keane and colleagues.^14^

The study is limited in that practitioners were all recruited from the same geographical region in one area of the UK. However, by conducting focus groups with a total of 21 primary care practitioners across both GP and PN roles, a wide variety of practices and experiences of managing patient use of blood pressure monitors at home have been elicited. Inclusion of healthcare assistants and practice managers in the focus groups may have shed more light on how self-monitoring fits within the wider structure of primary care. Use of vignettes may have led participant conversations, although it is considered this was necessary in order to stimulate discussion of the topics examined in the research.

The thematic analysis was limited in that it was principally completed by one researcher (JA); however, the analysis of one of the transcripts by three researchers, and the subsequent discussion of themes arising, ensured that themes were robust.

### Comparison with existing literature

Smolen and colleagues’ conceptual model of providers’ approach to hypertension management details three particular provider actions for successful blood pressure management: relationship building; self-management tools; and patient education.^9^ The findings provide detail in the ways that these provider actions are currently undertaken, highlighting variation in practice occurring among surgeries. For example, the findings indicate that healthcare assistants are increasingly providing support for home monitoring, which GPs and PNs may not have time to provide. Healthcare assistants may be more likely to understand local patient culture and provide a sense of continuity of care,^11^ which contributes to aspects of relationship building. Their role in supporting home monitoring could, therefore, bring benefits to patient care, beyond time-saving for GPs.

While prior research in this area has primarily focused on care occurring after a patient has obtained a home blood pressure monitor,^8^ the results show that there are important considerations to be made around whether and how patients obtain a monitor in the first place. Primary care professionals were found to advise patients differently on where to purchase a blood pressure monitor, including from pharmacies and online, and patients on low incomes were given other suggestions about how they might obtain a monitor. Thus, while Smolen and colleagues highlight the importance of self-management tools,^9^ the participants in the present study gave examples of how they guide patients to obtain these tools, taking account of differences across socioeconomic backgrounds.

While patients are known to turn to the internet to seek guidance for home monitoring of their own volition,^4^ the results demonstrate how primary care professionals advise patients to seek out online materials from sources, including YouTube, professional associations and NHS Choices (now referred to as the NHS website), and may send them direct links to online information. Thus, the study demonstrates the kinds of resources primary care professionals draw on and the way that these are disseminated to patients in the third part of Smolen *et al* *’s* conceptual model, patient education.

The findings also highlight how primary care professionals experience patient anxiety around self-monitoring, including through the use of ambulatory monitors. Anxiety around self-monitoring is recognised in guidance for patients with hypertension from the British Heart Foundation, which suggests that self-monitoring is *'* *not a good idea for everyone* *'* and that *'* *some people feel more anxious when taking their own blood pressure* *'*,^20^ although some research suggests that anxiety does not increase in those self-monitoring blood pressure.^21^ Here, the authors add that primary care professionals share guidelines on the frequency of monitoring and encourage patients to take a longer term view of blood pressure in order to mitigate patient concerns and worry.

### Implications for research and practice

This article has explored the experience of blood pressure self-monitoring in primary care. It has been demonstrated how pharmacists are called on to supply and support the use of blood pressure monitors by patients with hypertension, supporting suggestions in the Topol Review that the role of the pharmacist will need to evolve to include the supply and support of multiple healthcare technologies as the use of these increases.^22^ The findings show too that clinicians are finding workarounds to enable patients to obtain blood pressure monitors in more deprived areas. The NHS Long Term Plan promises greater funding for poorer areas,^23^ which could provide self-monitoring technologies to those who cannot afford them, without the need for such workarounds.

The results provide a view of how primary care practitioners currently manage the use of home blood pressure monitoring, and provide examples of practice that may be informative for those working in this area. In particular, a need to consider five aspects of patient self-monitoring of blood pressure (advising on purchase, supporting monitoring, mitigating potential anxiety, valuing patient data, and considering socioeconomic barriers) is highlighted. These findings are of particular importance since NICE guidance^6^ currently advocates that practitioners provide guidance and education to their patients on blood pressure monitoring, but lacks detail on how this should be conducted. The five aspects of managing self-monitoring that have been highlighted may also be useful when considering how primary care practitioners can best support self-monitoring by patients with conditions other than hypertension. The importance of the role of the healthcare assistant in helping patients to manage a self-monitoring practice is emphasised. It is also highlighted that there is a need to ensure patients are clear about how their data is used to encourage continued monitoring.

Further research is required to understand views of pharmacists and healthcare assistants on spending more of their time supporting patients with self-monitoring technologies, particularly as the number of self-monitoring technologies implemented in primary care for different conditions increases. Further work could also examine the potential of a standardised NHS care pathway for self-monitoring, which could include detail on how devices should be obtained, how their accuracy can be checked, target readings, when readings should be taken, and which healthcare professional should be consulted once readings are collated.
